# Aldehyde Dehydrogenase 1B1 Is Associated with Altered Cell Morphology, Proliferation, Migration and Chemosensitivity in Human Colorectal Adenocarcinoma Cells

**DOI:** 10.3390/biomedicines9010044

**Published:** 2021-01-06

**Authors:** Ilias Tsochantaridis, Angelos Roupas, Georgia-Persephoni Voulgaridou, Alexandra Giatromanolaki, Michael I. Koukourakis, Mihalis I. Panayiotidis, Aglaia Pappa

**Affiliations:** 1 Department of Molecular Biology & Genetics, Democritus University of Thrace, 68100 Alexandroupolis, Greece; iliatsoc@gmail.com (I.T.); aggelosrou@gmail.com (A.R.); georgiavou_85@hotmail.com (G.-P.V.); 2 Department of Pathology, University General Hospital of Alexandroupolis, Democritus University of Thrace, 68100 Alexandroupolis, Greece; agiatrom@med.duth.gr; 3 Radiotherapy/Oncology, Radiobiology & Radiopathology Unit, Department of Medicine, School of Health Sciences, Democritus University of Thrace, 68100 Alexandroupolis, Greece; targ@her.forthnet.gr; 4 Department of Electron Microscopy & Molecular Pathology, The Cyprus Institute of Neurology & Genetics, Nicosia 2371, Cyprus; mihalisp@cing.ac.cy; 5 The Cyprus School of Molecular Medicine, The Cyprus Institute of Neurology & Genetics, Nicosia 1683, Cyprus; 6 Department of Applied Sciences, Northumbria University, Newcastle Upon Tyne NE1 8ST, UK

**Keywords:** aldehyde dehydrogenase, ALDH, ALDH1B1, HT29, cell proliferation, cell morphology, chemoresistance, cell cycle, migration, cancer

## Abstract

Aldehyde dehydrogenases (ALDHs) are NAD(P)^+^-dependent enzymes that catalyze the oxidation of endogenous and exogenous aldehydes to their corresponding carboxylic acids. ALDHs participate in a variety of cellular mechanisms, such as metabolism, cell proliferation and apoptosis, as well as differentiation and stemness. Over the last few years, ALDHs have emerged as cancer stem cell markers in a wide spectrum of solid tumors and hematological malignancies. In this study, the pathophysiological role of ALDH1B1 in human colorectal adenocarcinoma was investigated. Human colon cancer HT29 cells were stably transfected either with human green fluorescent protein (GFP)-tagged ALDH1B1 or with an empty lentiviral expression vector. The overexpression of ALDH1B1 was correlated with altered cell morphology, decreased proliferation rate and reduced clonogenic efficiency. Additionally, ALDH1B1 triggered a G2/M arrest at 24 h post-cell synchronization, probably through p53 and p21 upregulation. Furthermore, ALDH1B1-overexpressing HT29 cells exhibited enhanced resistance against doxorubicin, fluorouracil (5-FU) and etoposide. Finally, ALDH1B1 induced increased migratory potential and displayed epithelial–mesenchymal transition (EMT) through the upregulation of *ZEB1* and *vimentin* and the consequent downregulation of *E-cadherin.* Taken together, ALDH1B1 confers alterations in the cell morphology, cell cycle progression and gene expression, accompanied by significant changes in the chemosensitivity and migratory potential of HT29 cells, underlying its potential significance in cancer progression.

## 1. Introduction

The aldehyde dehydrogenase (ALDH) superfamily consists of a variety of NAD(P)^+^-dependent enzymes that oxidize endogenous and exogenous aldehydes to their corresponding carboxylic acids. To date, 19 human ALDH isoenzymes have been characterized and categorized to 11 families and four subfamilies [1]. ALDHs are important cytoprotective enzymes, considering their metabolic role; however, they have also been correlated with additional homeostatic functions, like stemness, differentiation, cell proliferation, tumorigenesis and retinoic acid signaling [2,3,4,5,6,7]. For instance, members of the ALDH1 and ALDH3 families protect the lens of the eye by detoxifying aldehydes produced by ultraviolet radiation [8], oxidizing aldophosphamide and detoxifying oxazaphosphorines [9,10], while their transcription is related to drug resistance [11]. Interestingly, several ALDH isoforms have also been characterized as markers of normal and cancer stem cells (CSCs) [12,13]. CSCs exhibit stem-related properties, such as a self-renewal ability, increased clonogenicity/tumorgenicity and enhanced chemo-/radio-resistance, thereby potentially driving cancer progression and metastasis [12,13,14,15,16,17,18,19]. Indeed, ALDH expression have been associated with several CSC-related features, like chemo- and radio-resistance [3,9,20,21,22,23], hypoxia [6,24,25], cell proliferation and invasion [3,26,27].

ALDH1B1 is a mitochondrial NAD^+^-dependent enzyme that metabolizes acetaldehyde, 4-hydroxynonenal (4-HNE), malondialdehyde (MDA) [28], nitroglycerin and retinaldehyde [29]. Still, little is known about its physiological and pathophysiological roles, but several studies have indicated its association with colorectal [30,31,32,33], pancreatic [34,35], osteosarcoma [4], liver [36], gastric [37] and lung [38] tumorigenesis. However, its exact role in carcinogenesis and cancer progression remains elusive. In this study, we investigated the role of ALDH1B1 in the pathophysiology of human colon adenocarcinoma. To this end, we utilized the human colon adenocarcinoma cell line (HT29) and generated an isogenic HT29 cell line pair (differing only in the expression of human GFP-tagged ALDH1B1 in order to investigate the effect of ALDH1B1 overexpression in (i) cell morphology, (ii) cell proliferation and colony formation efficacy, (iii) cell cycle progression, (iv) cell viability (under conditions of exogenous stresses imposed by treatment with chemotherapeutic agents), (v) gene expression profile of epithelial–mesenchymal transition (EMT) markers and (vi) cell migration.

## 2. Experimental Section

### 2.1. Materials

Cell lines were obtained from American Type Culture Collection (ATCC, Manassas, VA, USA). Culture medium, fetal bovine serum (FBS), penicillin/streptomycin (100X) solution and trypsin were from Biosera (Boussens, France). Lipofectamine 2000 was purchased from Invitrogen (Thermo Fisher Scientific, Waltham, MA, USA), and puromycin was from Clontech (Mountain View, CA, USA). The human ALDH1B1 clone in the pCMV6-XL4 vector (SC119705) and the lentiviral vectors pLenti-C-mGFP-P2A-Puro (PS100093) and pLenti-P2A-Puro (PS100109) were obtained from Origene (Rockville, MD, USA). Co-vectors for lentiviral transduction pCMVR8.74 and pCMV-VSV-G were obtained from Addgene (Watertown, MA, USA). Propidium iodide (PI) and 4′,6-diamidino-2-phenylindole (DAPI) were purchased from Biotium (Landing Parkway Fremont, CA, USA). Fluoromount-G™ from Thermo Fisher Scientific (Waltham, MA, USA) and sulforhodamine B (SRB) dye and all chemotherapeutic agents were obtained from Sigma-Aldrich (St. Louis, MO, USA). For protein estimation, the Pierce™ BCA Protein assay was purchased from Thermo Fisher Scientific (Rockford, IL, USA), while polyvinylidene difluoride (PVDF) membranes and chemiluminescence reagents were obtained from Millipore (Bedford, MA, USA). Primary antibodies anti-p53 (1C12) and anti-p21 (12D1) were obtained from Cell Signaling Technology (Boston, MA, USA), whereas anti-β actin was from Novus Biologicals (Centennial, CO, USA). Goat anti-rabbit and anti-mouse immunoglobulin G (IgG) horseradish peroxidase conjugated antibodies were purchased from Millipore (Bedford, MA, USA), while goat anti-rabbit (for flow cytometry) was from Jackson Immunoresearch (Cambridge House, St. Thomas Place, Cambridgeshire, UK). NucleoZOL was obtained from Macherey-Nagel (Düren, Germany), whereas all primers, dNTPs, random hexamers and PrimeScript reverse transcriptase from Invitrogen (Thermo Fisher Scientific, Waltham, MA, USA). Finally, the KAPA SYBR Fast Master Mix solution was purchased from Kapa Biosystems (Hoffmann-La Roche, Basel, Switzerland).

### 2.2. Cell Culture

Human colon adenocarcinoma (HT29 and Caco2) and human embryonic kidney (HEK) 293 cells were used in the study. All cell lines were maintained in Dulbecco’s modified Eagle’s medium (DMEM) (DMEM with high glucose, for HT29 and HEK-293 and DMEM/nutrient mixture F-12 for Caco2) supplemented with 10% FBS and 1X penicillin/streptomycin solution. Cells were cultured at 37 °C with 5% CO_2_ in a humidified incubator.

### 2.3. Lentiviral Transduction

The ALDH1B1 cDNA was subcloned from pCMV6-XL4/ALDH1B1 into the pLenti-C-mGFP-P2A-Puro lentiviral gene expression vector. Human embryonic kidney (HEK) 293 cells (ATCC) (3 × 10^5^) were seeded in 6-well plates and, the next day, were transfected with 1 μg ALDH1B1-GFP/vector or the vector alone (pLenti-P2A-Puro) with 1 μg pCMVR8.74 and 0.5 μg pCMV-VSV-G using the Lipofectamine 2000 reagent. HT29 or Caco2 cells (2.5 × 10^5^) were seeded in another 6-well plate, and after a day (48 h post-transfection), the supernatant (S/N) culture from HEK 293 cells was collected. Then, HT29 or Caco2 cells were infected with 300 μL S/N in 200 μL DMEM complete medium. Every 10 min, the plate was shaken for 30 min, and then, 500 μL DMEM complete medium was added. After 2 h, S/N culture was removed, and 1 to 2 mL DMEM complete medium was added. Stably transfected cells were selected in the presence of 1 μg/mL of puromycin (5–7 days) in the culture medium 48 h post-infection. Selected cells were propagated in the presence of puromycin.

### 2.4. Flow Cytometry Analysis

#### 2.4.1. Transfection Efficiency

PBS-washed HT29/mock or Caco2/mock and HT29/ALDH1B1 or Caco2/ALDH1B1 cells were stained with PI for 3 min, placed into fluorescence-activated cell sorting (FACS) tubes (BD Biosciences, Franklin Lakes, NJ, USA) and analyzed in an Attune NxT flow cytometer (Thermo Fisher Scientific). Transfection efficiency of HT29 or Caco2 cells carrying the ALDH1B1 gene was assessed as the percentage of GFP-stained cells (HT29/ALDH1B1 or Caco2/ALDH1B1) compared to control (HT29/mock or Caco2/mock) cells.

#### 2.4.2. Flow Cytometric Analysis of Cell Size and p21 Expression

PBS-washed cells were analyzed in the Attune NxT flow cytometer and Flowjo software (FlowJo LLC, Ashland, OR, USA) was used to generate histograms of forward and side scatter (FCS and SCS respectively) for HT29/mock or Caco2/mock and HT29/ALDH1B1 or Caco2/ALDH1B1 cells. We evaluated the cell morphology according to the established relationship between FSC and SSC, cell size, granularity and cell surface topography [39]. The median fluorescence intensity values of FSC and SSC were utilized as indicators for the quantification of the morphological differences between HT29/mock or Caco2/mock and HT29/ALDH1B1 or Caco2/ALDH1B1 cells.

PBS-washed cells were fixed with 4% formaldehyde, permeabilized with ice-cold 100% methanol, immunostained with primary and secondary antibodies and, finally, analyzed on the Attune NxT flow cytometer. Histograms of p21 median fluorescence intensity were generated by Flowjo software (FlowJo LLC, Ashland, OR, USA).

#### 2.4.3. Cell Counting and Growth Rate Determination

Cell counting was performed by flow cytometry. In brief, HT29/mock and HT29/ALDH1B1 (4 × 10^5^) cells were seeded in 10 cm culture dishes. Then, cells were trypsinized, washed with PBS and counted every 24 h for 3 consecutive days. Growth rate of cell cultures represents the number of doublings occurring per day (estimated by using an online doubling time calculator; https://doubling-time.com/compute.php).

#### 2.4.4. Cell Cycle Analysis

HT29/mock and HT29/ALDH1B1 cells (1.5 × 10^6^) were seeded in 10 cm culture dishes and, subsequently, were placed in a humidified incubator (37 °C, 5% CO_2_). Next day, the complete culture medium was removed, and cells were incubated for 24 h in serum-free culture medium to get synchronized. Then, PBS-washed cells were fixed in ice-cold 75% ethanol and incubated for at least 1 day at −20 °C. The cells were counted, stained with propidium iodide (50 μg/mL), incubated for 40 min in the dark and, finally, analyzed in a flow cytometer. The same process was performed for 12, 24 and 36 h post-synchronization samples.

### 2.5. Real-Time PCR

Total RNA was extracted by utilizing the NucleoZOL reagent according to the manufacturer’s instructions. For cDNA synthesis, 4 μg of total RNA and SuperScript™ First-Strand Synthesis kits were used according to the manufacturer’s instructions. Real-time PCR was performed using KAPA SYBR Fast Master Mix according to the manufacturer’s instructions. Reactions were performed by using the Applied Biosystems Step One instrument (Thermo Fisher Scientific, Waltham, MA, USA). The sequences of these primers are presented in Table 1. Reactions were run as triplicates in three independent experiments. Gene expression was normalized to β-actin using the 2^−ΔΔCT^ method.

### 2.6. Fluorescence Microscopy

Cells (2.5 × 10^5^) were plated on the surface of coverslips and, after 24 h, were fixed with 4% formaldehyde in 1X PBS for 20 min and then washed with 1X PBS (x3). Following a neutralization step with the addition of 1 M of glycine (pH 8.5), nuclei were counterstained with 4’-6-diamidino-2-phenylindole (DAPI) (1 μg/mL). Cells were washed with 1X PBS (x3), then mounted with Fluoromount-G™ (Thermo Fisher Scientific, Waltham, MA, USA) and, finally, processed for observation on a Nikon ECLIPSE E200 fluorescence microscope under 40X and 100X lenses. Images of cells were captured, and pictures were analyzed by image analysis software (ImageJ; National Institute of Health (NIH), Bethesda, MD, USA).

### 2.7. Colony Formation Assay

Cells (10^3^) were plated in 10 cm culture dishes and grown in a humidified incubator (37 °C, 5% CO_2_). Plates were monitored every 2 to 3 days for the formation of visible colonies (approximately 15–20 days). Fixing and staining of the cells were performed with the use of 0.5% (*w*/*v*) of crystal violet solution diluted in 25% methanol. Colonies (containing ≥50 cells) were counted using a stereomicroscope, whereas digital images were captured and processed using ImageJ software (NIH, Bethesda, MD, USA).

### 2.8. Sulforhodamine B (SRB) Assay

The SRB assay was performed as described previously [40]. Briefly, 4 × 10^3^ HT29 cells per well were cultured in 96-well plates. After a day, cells were incubated with increasing concentrations of different chemotherapeutic agents for 48 h (etoposide, 0–500 μM) or 72 h (doxorubicin, 0–5 μM and fluorouracil (5-FU), 0–100 μM) while fixed by ice-cold trichloroacetic acid (TCA) and then stained with SRB dye in 1% (*v*/*v*) acetic acid. Finally, the bound dye was dissolved in a 10-mM Tris base, and the absorbance was measured at 570 nm by a multi-plate reader (Tecan, Mannedorf, Switzerland). The percent (%) cell viability was calculated using the formula

[(Sample OD_570_ − media blank OD_570_)]/[(mean control OD_570_ − media blank OD_570_)] × 100 (1)

The EC_50_ (effective concentration of inducing a 50% decrease in cell viability) values were calculated by Sigma Plot Software v.10 (Systat, San Jose, CA, USA) via a four-parameter logistic curve.

### 2.9. Western Immunoblotting

Western immunoblotting was performed as described previously with minor modifications [41]. PBS-washed cells were lysed with radioimmunoprecipitation assay (RIPA) lysis buffer (50 mM sodium chloride, 1% Nonidet P-40 (NP-40), 0.25% sodium deoxycholate, 0.25% sodium dodecyl sulfate (SDS), 50-mM Tris, pH 8.0 and 1-mM ethylenediaminetetraacetic acid (EDTA)), supplemented with protease and phosphatase inhibitors. Cell lysates were incubated at 4 °C for 30 min, vortexed every 10 min and then centrifuged at 12,500 rpm for 15 min. Approximately, forty (40) μg whole-cell fractions were prepared and subjected to SDS-PAGE on 10% Tris-Glycine gels. Separated proteins were transferred to PVDF membranes, blocked with 5% nonfat dry milk in Tris-buffered saline with 0.1% Tween-20 detergent (TBST) at room temperature (RT) for 2 h and hybridized overnight at 4 °C with primary antibodies against anti-p53 (1/1000) and anti-β actin (1/5000). Then, the membranes were incubated with secondary horseradish peroxidase conjugated anti-rabbit and anti-mouse IgG antibodies (1/5000) for 1 h at RT, and immunoblot bands were developed by utilizing a Chemidoc MD Imaging System (Bio-Rad, Hercules, CA, USA). The stripping process was performed as described previously [41].

### 2.10. Sphere-Formation Assay

The sphere-formation assay was performed as described previously with minor modifications [42,43]. In brief, HT29 cells were enzymatically dissociated in trypsin/EDTA (1X), washed with PBS (1X) and then seeded at a clonal density of 10 cells/μL in a serum-free medium (DMEM/F-12; 3:1 mixture) containing 1X B-27 supplement, 0.4% (*w*/*v*) bovine serum albumin (BSA), 10-ng/mL recombinant epidermal growth factor (EGF), 20-ng/mL recombinant bovine fibroblast growth factor (bFGF) and 5-μg/mL insulin in T75 flasks. Spheres were formed after 8–12 days by changing culture medium every 2 to 3 days. First-generation spheres were used in all subsequent experiments.

### 2.11. Scratch Assay

HT29/mock and HT29/ALDH1B1 (7.5 × 10^5^) cells were seeded in 6-well plates in complete culture medium. After overnight incubation (at 37 °C and 5% CO_2_), cells were incubated for 24 h in serum-free culture medium to get synchronized. Then, the serum-free medium was removed, and cells were washed twice with PBS and then scratched with a pipette tip. Cells were photographed at indicated time points with a ZEISS Primovert light microscope (Zeiss, Göttingen, Germany) equipped with a digital camera (Axiocam ERc 5 s). For each time point, multiple photographs were analyzed by ImageJ software, and the average % wound area (% open image area) was calculated.

### 2.12. Statistical Analysis

For all statistical analyses, values were expressed as mean ± SD by utilizing GraphPad Prism software, version 8.3.0 (San Diego, CA, USA). A Student’s *t*-test was used in order to compare results between two groups. On the other hand, analysis of two variables among multiple groups was performed by using a two-way ANOVA followed by Tukey’s multiple comparison tests. A value of *p* < 0.05 was considered statistically significant, and at least three independent experiments were performed under each experimental design.

## 3. Results

### 3.1. Generation and Characterization of the HT29 Isogenic Cell Line Pair

**Figure 1:**
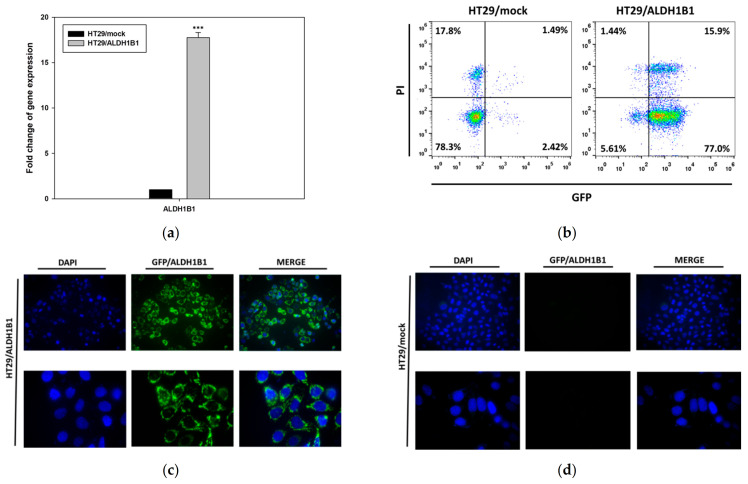
Expression of *ALDH1B1* in the HT29 isogenic cell line pair. (**a**) *ALDH1B1* gene expression levels detected by real-time PCR in HT29/mock and HT29/ALDH1B1 cells. (**b**) Evaluation of GFP+ cells (green) in HT29/mock and HT29/ALDH1B1 by flow cytometry analysis. Nuclei were stained with propidium iodide (PI) (red). Fluorescence microscopy for detecting the GFP+ cells (green) in HT29/mock (**c**) and HT29/ALDH1B1 (**d**) cells (upper panel at 40× magnification and lower panel at 100× magnification). Nuclei were stained with DAPI (4′,6-diamino-2-phenylindole) (blue). Results are expressed as mean ± SD of three independent experiments. *** *p* < 0.001.

Stable transfection of the human ALDH1B1 cDNA in HT29 cells generated the polyclonal HT29/ALDH1B1 cell line (Figure 1). First, the ALDH1B1 expression was evaluated by real-time quantitative PCR, and the transcriptional levels of *ALDH1B1* were found to be >17-fold higher than those in HT29/mock cells (Figure 1a). Furthermore, the expression of GFP-tagged ALDH1B1 was confirmed by flow cytometry. In HT29/ALDH1B1 cells, 92.9% were GFP+ (15.9% were PI+), while, in HT29/mock cells, 96.1% were GFP− (17.8% were PI+) (Figure 1b). Regular monitoring of the ALDH1B1 mRNA levels, together with GFP tag fluorescence, confirmed the maintenance of stable ALDH1B1 expression. To further validate the transfection, we assessed the GFP fluorescence signal by fluorescence microscopy. Indeed, Figure 1c,d depicts the enhanced GFP fluorescence in HT29/ALDH1B1 versus HT29/mock cells, respectively.

**Figure 2:**
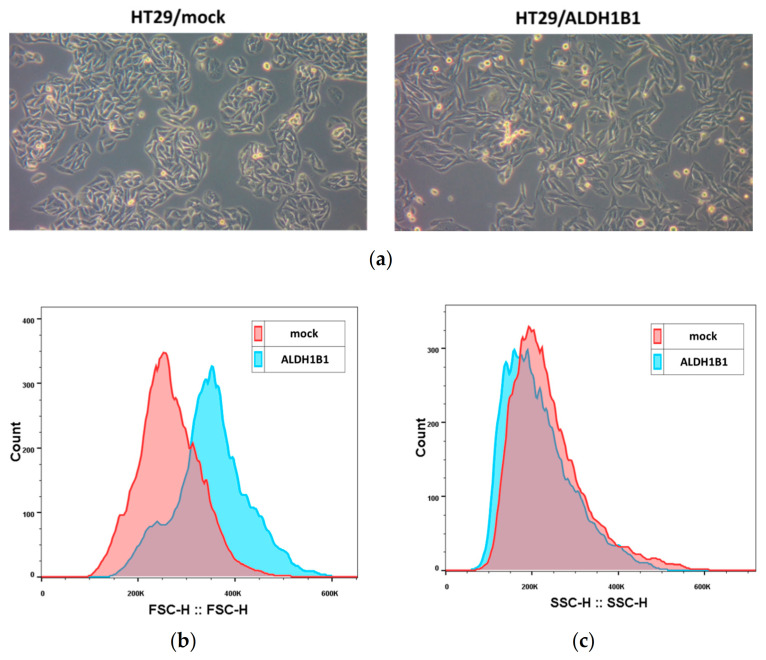
Expression of ALDH1B1 triggers significant morphological alterations in HT29 cells. (**a**) Photographs of the HT29 isogenic cell line pair demonstrating the morphological differences under optical microscopy (magnification 20×). ALDH1B1-overexpressing cells have different sizes (**b**) and granularity (**c**) compared to mock control cells. At least 20,000 events were analyzed through flow cytometry, and the median fluorescence intensity of forward scatter (FCS) and side scatter (SSC) parameters was determined in both cell lines (Table 2). Representative histograms of FSC (**b**) and SSC (**c**) of the HT29 isogenic cell line pair. The HT29/ALDH1B1 histogram (light blue color) had appreciably right-shifted forward and left-shifted side scatter, demonstrating the bigger size and lower granularity of ALDH1B1-overexpressing cells in comparison to HT29/mock cells (pink color). Graphs are representative of three independent experiments performed under each experimental condition.

In addition, we observed that the expression of ALDH1B1 triggered significant morphological differences in HT29 cells. More specifically, ALDH1B1-overexpressing cells appeared more elongated, with lower clonogenic ability, compared to mock control cells (Figure 2a). By utilizing an established methodology for assessing cell morphology [39], both cell lines were analyzed through flow cytometer and by means of FSC and SSC indices. All generated histograms for FSC (Figure 2b) and SSC (Figure 2c) indicated increased cell sizes and lower internal structural complexity of HT29/ALDH1B1 in comparison to HT29/mock cells. The median fluorescence intensity values for FSC and SSC are also shown in Table 2.

### 3.2. ALDH1B1 Is Related to Decreased Cell Proliferation and Clonogenicity and Induces G2/M Arrest through p53 Upregulation in HT29 Cells

**Figure 3:**
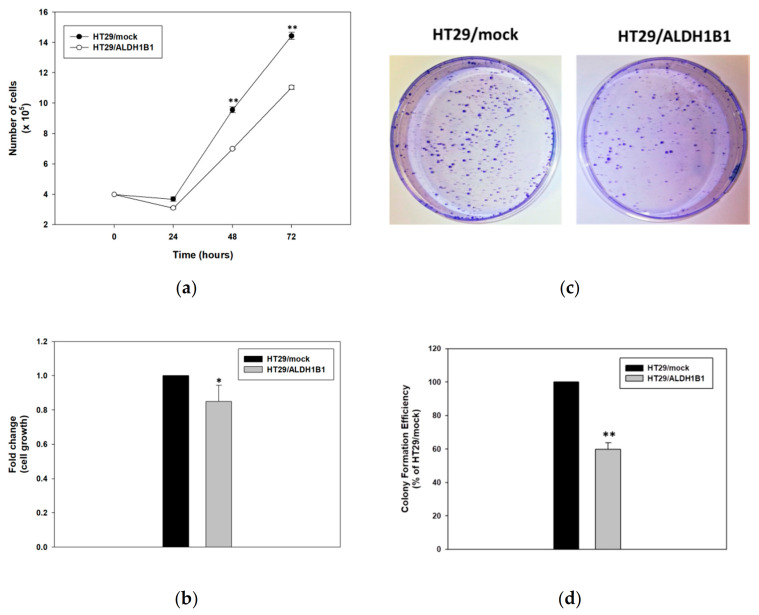
Expression of ALDH1B1 decreases the cell proliferation rate and clonogenic efficiency in HT29 cells. (**a**) Growth rates of HT29/mock and HT29/ALDH1B1 cells. Cells were plated at a density of 4 × 10^5^ cells/plate in 10-cm culture plates, and the total number of cells was determined every 24 h over 3 consecutive days. (**b**) Confluency of HT29/ALDH1B1 cells versus HT29/mock cells at 72 h post-seeding. Cells were seeded at a density of 4 × 10^3^ cells/well in 96-well plates, grown for 72 h and processed for fixation and staining by the sulforhodamine B (SRB) assay. Absorbance was measured at 570 nm, and the cell viability percentage (%) was calculated and normalized relative to the control (HT29/mock) cells. (**c**) Representative plates of colony formation efficiency of HT29/mock and HT29/ALDH1B1 cells. Cells (1000) were seeded in 10-cm culture plates and observed for colony formation approximately 20 days following their plating. Then, cells were stained with crystal violet. (**d**) Quantitative assessment of colony formation efficiency. Results are expressed as mean ± SD of three independent experiments. * *p* < 0.05 and ** *p* < 0.01.

Next, we investigated the effect of ALDH1B1 in the proliferation rate of HT29 cells. Specifically, HT29/ALDH1B1cells exhibited lower growth rates in comparison to HT29/mock ones (Figure 3a,b). The isogenic cell line pair was seeded and counted at four different time points (0, 24, 48 and 72 h). The number of cells was statistically significantly lower in HT29/ALDH1B1 compared to HT29/mock ones (Figure 3a). In fact, the doubling time (tD) of the HT29/ALDH1B1 cells was lower, while the growth rates (GR) were higher than the corresponding tD and GR of the HT29/mock cells (Table 3). Similarly, it was observed that TCA-fixed and SRB-stained HT29/ALDH1B1 cells had considerably lower growth capacity compared to mock control cells (Figure 3b). Moreover, the counted colonies of ALDH1B1-overexpressing cells were substantially fewer compared to mock cells, demonstrating that ALDH1B1 may affect the clonogenicity of HT29 cells (Figure 3c). Finally, the estimated colony formation efficiency of HT29/ALDH1B1 was approximately 58% ± 3.93% that of the control (mock) cells (Figure 3d). Similar effects of ALDH1B1 on cell proliferation, clonogenicity and morphological alterations were observed when a different colorectal cell line, CaCo2, was used (Supplementary Figure S1a–d and Table S1).

**Figure 4:**
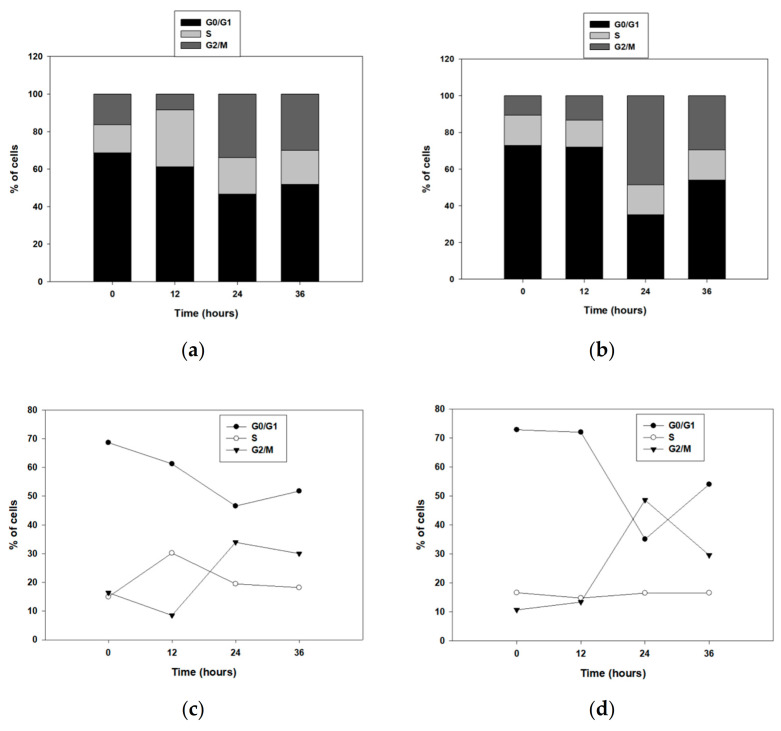

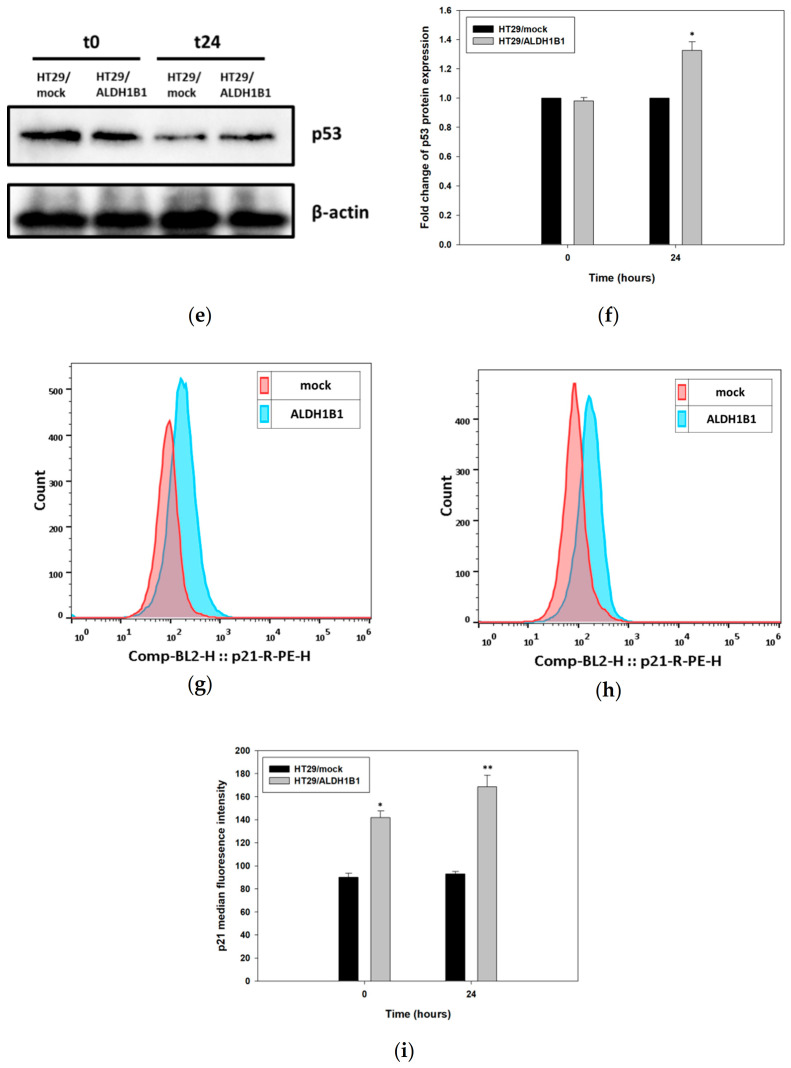
ALDH1B1 induces G2/M cell cycle arrest 24 h post-synchronization through p53-dependent upregulation. (**a**–**d**) Cell cycle kinetics in the HT29 isogenic cell line pair. Cells were synchronized in the G0/G1 phase (starvation, 24 h), and cell cycle kinetics were analyzed at the indicated time points. Approximately, 20% more HT29/ALDH1B1 cells were found in the G2/M phase 24 h post-synchronization compared to HT29/mock cells. The upregulation of p53 (>1.3-fold higher than HT29/mock cells) 24 h post-synchronization (**e**,**f**) led to p21 upregulation in ALDH1B1-overexpressing cells (**g**–**i**). The ALDH1B1 histogram (light blue color) had appreciably right-shifted at the time of synchronization (**g**) and 24 h post-synchronization (**h**) in comparison with HT29/mock cells (pink color), indicating the increased p21 expression levels in ALDH1B1-overexpressing cells. At least 20,000 events were analyzed through flow cytometry, and the median fluorescence intensity for p21 was determined in both cell lines (Table 4). Equal protein loading was verified by stripping and re-probing the same membranes with β-actin (**e**). The above graphs are representative of an experiment. At least three independent experiments were performed for each condition. * *p* < 0.05 and ** *p* < 0.01.

Then, we studied the potential role of ALDH1B1 in the cell cycle by assessing the cell cycle kinetics (Figure 4). First, the HT29 isogenic cell line pair was seeded overnight and then incubated in serum-free medium (starvation) for 24 h to get synchronized in the G0/G1 phase. Cells were fixed in 75% ethanol, PI-stained at the indicated time points and then analyzed by flow cytometry. Interestingly, ALDH1B1 expression induced a significant G2/M growth arrest (Figure 4b,d). More than 20% of HT29/ALDH1B1 cells (Figure 4b,d) were at the G2/M phase when compared to the control (mock) cells (Figure 4a,c) over 24 h post-synchronization. Possibly, ALDH1B1-overexpressing cells were subjected to a G2/M growth arrest through a p53-dependent cell cycle pathway. In fact, it was demonstrated that p53 protein expression levels at 24 h post-synchronization were increased (1.3-fold) in HT29/ALDH1B1 when compared to HT29/mock cells (Figure 4e,f). Moreover, p53 upregulation resulted in p21 upregulation as well in ALDH1B1-overexpressing cells (Figure 4g–i). p21 is known to inhibit CDK1 and induce arrest at the G2/M transition cell cycle point [44]. Median fluorescence intensity for p21 is presented in Table 4.

### 3.3. Expression of ALDH1B1 Confers Resistance to Various Chemotherapeutic Agents in HT29 Cells

**Figure 5:**
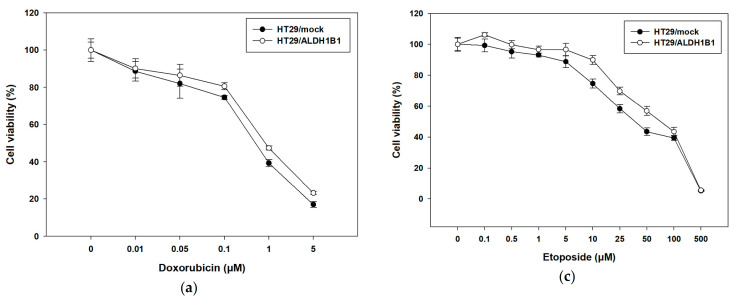

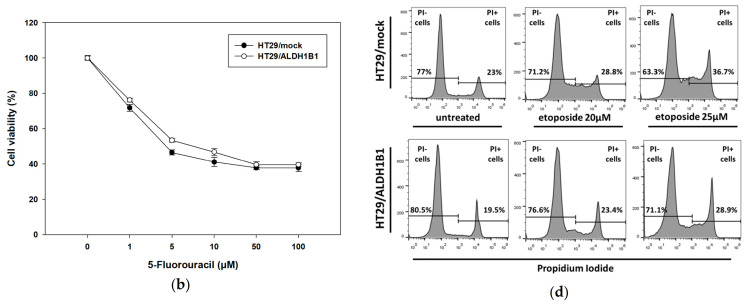
Effects of various chemotherapeutic agents on the cell viability of HT29/mock and HT29/ALDH1B1 cells. Viability curves of HT29/mock and HT29/ALDH1B1 cells after exposure to (**a**) doxorubicin, (**b**) 5-fluorouracil and (**c**) etoposide for 72 h (**a**,**b**) and 48 h (**c**). Viability curves of the HT29/ALDH1B1 cells are shifted to the right, indicating increased cellular tolerance to the cytotoxic effect of the agents. (**d**) Cells (1.5 × 10^6^) were seeded in 10-cm culture plates and treated with etoposide for 48 h. After washing, cells were stained with propidium iodide (PI) and analyzed by flow cytometry for the assessing % of dead cells. At least three independent experiments were performed under each experimental condition.

Next, we studied the response of this isogenic cell line pair to different chemotherapeutic and oxidative agents characterized by different modes of actions. HT29/mock and HT29/ALDH1B1 were treated with increasing concentrations of doxorubicin and 5-FU for 72 h and etoposide for 48 h. Cell viability curves were plotted (Figure 5), and EC_50_ values for each condition were determined (Table 5). Our data suggest that ALDH1B1 was related to a chemoresistant phenotype, as demonstrated by the viability curves in HT29/ALDH1B1 compared to the HT29/mock cells. ALDH1B1-overexpressing cells exhibited approximately two-fold resistance to doxorubicin (Figure 5a and Table 5) and 5-FU (Figure 5b and Table 5) and 1.5-fold to etoposide (Figure 5c and Table 5) in comparison with mock control cells. The increased etoposide resistance of HT29/ALDH1B1 cells was also confirmed through flow cytometry analysis. PI-stained (late apoptotic and dead) cells were significantly more in HT29/mock compared to HT29/ALDH1B1 under the treatments of two different concentrations (20 and 25 μM) of etoposide (Figure 5d and Table 6). No differences were observed between PI- and PI+ cells between HT29/mock and HT29/ALDH1B1 (Figure 5d and Table 6).

### 3.4. ALDH1B1 Promotes Migration and EMT through ZEB1 and Vimentin Upregulation in HT29 Cells

**Figure 6:**
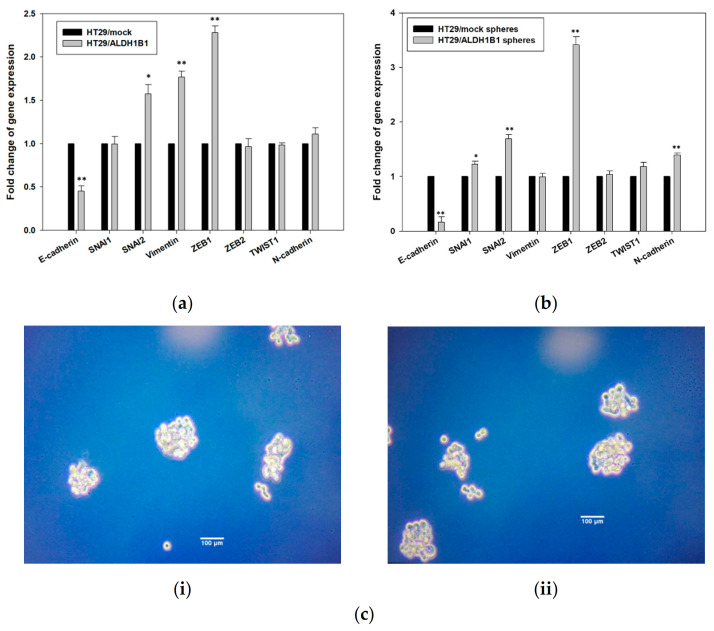
Expression of ALDH1B1 induces epithelial–mesenchymal transition (EMT) in HT29 cells and spheres. The effects of ALDH1B1 on the gene expression levels of EMT-related transcription factors in (**a**) differentiated and (**b**) cancer stem-like cells in the HT29 isogenic cell line pair. The comparative quantification ΔΔCt method was utilized for analyzing the fold change in the gene expression levels. β-actin gene was used as the endogenous control for the normalization of the samples. (**c**) Representative images of (**i**) HT29/mock and (**ii**) HT29/ALDH1B1 spheres (magnification 20×), which were typically formed in approximately 10 days. Results are shown as mean ± SD. At least three independent experiments were performed under each experimental condition. * *p* < 0.05 and ** *p* < 0.01.

The lower cell proliferation and morphological changes of ALDH1B1-overexpressing cells, along with their chemoresistant phenotype, prompted us to further evaluate if ALDH1B1 expression could be associated with any migratory effect and EMT transcriptional alternations in HT29 cells. Overall, cells, during the EMT process, proliferate at lower rates and may be subject to cytoskeletal changes [45]. EMT induction is also correlated with cancer stem-like characteristics, such as stemness and metastatic potential [46]. Therefore, we assessed the mRNA expression profile of several effector molecules and transcription factors of EMT. In general, EMT is associated with the disintegration of tumor cell junction and interruption of apical-basal polarity, thereby affecting the cytoskeletal architecture and promoting an invasive potential [47]. Consequently, we analyzed the gene expression profile of EMT-related molecules such as *E-cadherin*, *SNAI1*, *SNAI2*, *vimentin*, *ZEB1*, *ZEB2*, *TWIST1* and *N-cadherin* in the HT29 isogenic cell line pair. Our results demonstrated that ALDH1B1 induced the transcriptional levels of *ZEB1* and *vimentin*, resulting in the downregulation of *E-cadherin* (Figure 6a). Considering that the EMT process is connected with normal and cancer epithelial cells [48] (resulting in the generation of metastatic CSCs [49]), we also assessed the expression of EMT-related genes in HT29-generated spheres (Figure 6b,ci,ii). We confirmed that ALDH1B1 induces EMT through the upregulation of *vimentin* and *ZEB1* and subsequent downregulation of *E-cadherin* in HT29 cells. Similar results on *vimentin* upregulation and *E-cadherin* downregulation were observed in Caco2 cells transfected with human ALDH1B1 (Figure S1e).

**Figure 7:**
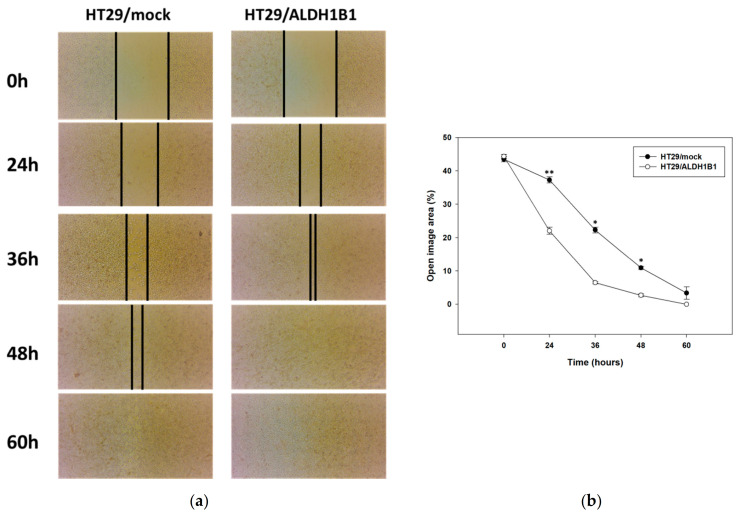
ALDH1B1 is related to the increased migratory potential in HT29 cells. (**a**) Scratch assay for the HT29 isogenic cell line pair. At the indicated time points, cell migration was monitored under an optical microscope (magnification 20×). (**b**) Quantification of the percentage of wound closure by ImageJ software analysis in HT29 cells. Data are presented as mean ± SD of three independent experiments. * *p* < 0.05 and ** *p* < 0.01.

Finally, we determined the migratory potential of the isogenic cell line pair by employing the scratch assay. Our results showed that HT29/ALDH1B1 cells migrated faster than HT29/mock cells, as indicated in Figure 7a. The percentage of wound closure for ALDH1B1-overexpressing cells was approximately two-fold lower compared to control (mock) cells, demonstrating the increased migratory potential of ALDH1B1-overexpressing cells (Figure 7b).

## 4. Discussion

Aldehyde dehydrogenase 1B1 is a member of the multifunctional ALDH superfamily, which is mainly localized in the mitochondria. To date, ALDH1B1 has no clear physiological and pathophysiological roles; however, recent studies have contributed to the better functional characterization of that enzyme. Specifically, ALDH1B1 has been found to be involved in β-cell development (in mice) [50], the maintenance of sperm motility (in horses) [51] and ethanol and retinaldehyde metabolism (in humans) [28]. Further studies have demonstrated that ALDH1B1 is associated with diabetes [52], colon cancer [30,31,32,33], pancreatic cancer [34,35] and osteosarcoma [4], as well as in regulating different CSC-related signaling pathways, such as PI3K/Akt, Notch and Wnt/β-catenin [30]. The overexpression of ALDHs is involved in tumor progression through CSCs [53], giving cancer cells a survival advantage against oxidative damage, lipid peroxidation and toxic aldehydes, all of which are involved in slowing down cell proliferation [54].

The main aim of our study was to explore the potential effect of ALDH1B1 in human colon adenocarcinoma. Therefore, we generated an isogenic HT29 cell line pair, differing only in the expression of GFP-tagged ALDH1B1. Interestingly, the expression of ALDH1B1, in HT29 cells, was correlated with altered cell morphology, a lower cell proliferation rate and clonogenic efficiency, as well as G2/M-induced p53-dependent cell cycle growth arrest. Moreover, ALDH1B1 conferred an enhanced chemoresistance to HT29 cells against different chemotherapeutic agents like doxorubicin, 5-FU and etoposide. We also confirmed EMT induction in the ALDH1B1-overexpressing cells (as well as their spheres) through *ZEB1* upregulation and increased migratory potential.

Although little is known about ALDH1 in relation to cellular morphological changes, there is evidence supporting an association of ALDH1 with breast CSC and retinoid signaling [55], in which retinoic acid can induce crucial cellular alterations such as differentiation, cell cycle growth arrest and morphological changes [56]. As demonstrated in Figure 2a, ALDH1B1-overexpressing HT29 cells acquired a more elongated shape compared to HT29/mock cells, probably due to the induction of EMT, in which cells have increased migratory potential and vimentin area, triggering the elongation of the cell nucleus and cytoplasm [57,58]. Furthermore, HT29/ALDH1B1 cells displayed slower proliferation rates than HT29/mock cells and decreased the colony-forming ability (Figure 3 and Table 3). The overexpression of ALDH1B1 caused statistically significant p53-dependent G2/M growth arrest at 24 h post-synchronization compared to mock control cells (Figure 4). A similar association of ALDH expression and p53 upregulation was recently demonstrated by the expression of ALDH3A1 in human corneal epithelial cells (HCE-2 cells), which resulted in transcriptional upregulation of the p53 protein. This protein (p53) induces the transcription of p21 (by binding to two sites of its promoter), thereby causing the inhibition of cyclin B/Cdc2 complex activity and, eventually, cell cycle growth arrest at the G2/M phase [44], which can further explain the slower proliferation rate of ALDH1B1-overexpressing cells. Other studies have also documented the effects of ALDH1 family members in the cell processes of proliferation, invasion and migration. When the expression of ALDH1A3 was suppressed in human cancer cell lines, an opposite effect on cell proliferation and invasion was observed and associated with the differential expression of the CXC chemokine receptor 4 [26]. ALDH1 expression was correlated with the properties of cancer stem cells in cervical carcinoma, and ALDH-positive cells displayed significantly higher rates of cell proliferation, microsphere formation and migration [59]. ALDH1B1, apart from being a potential colon cancer biomarker, was also shown to be crucial for tumor development by modulating canonical Wnt/β-catenin, Notch and PI3K/Akt signaling pathways [30].

On another note, ALDH1B1-overexpressing cells exhibited an enhanced tolerance against chemotherapeutic agents like 5-FU, doxorubicin and etoposide. Our results are in line with previous studies showing that ALDH2- and ALDH1A2-expressing K562 and H1299 cells exhibited increased resistance against 4-hydroperoxy cyclophosphamide and doxorubicin [60]. Noteworthy, ALDH+ cells (with a stem-cell phenotype) were related to doxorubicin and etoposide resistance in Ewing sarcoma [61], while another study showed that the high activity of ALDH1 was associated with 5-FU resistance [62]. Similar observations were obtained with ALDH3A1+ cells exhibiting high tolerance against three different chemotherapeutics (e.g., doxorubicin, 5-FU and etoposide) in breast cancer Michigan Cancer Foundation-7 (MCF-7) cells [3]. The increased chemoresistance of HT29/ALDH1B1 prompted us to further investigate the effect of ALDH1B1 in EMT-related transcription factors and core regulators. In addition, the increased invasion ability and metastatic potential are also important features of CSCs, enabling them to migrate to other sites and initiate new tumors [63]. EMT is a process, responsible for forming CSCs [47] and related to CSC plasticity [64]. To this end, we first assessed the mRNA levels of various EMT mediators, such as *SNAI1, SNAI2, vimentin, ZEB1, ZEB2, TWIST1* and *N-* and *E-cadherin* in the isogenic cell line pair and in their generated spheres. We demonstrated that ALDH1B1 induced EMT in HT29 cells and cancer stem-like cells (through the upregulation of *ZEB1*), resulting in the downregulation of *E-cadherin*. We suggest that ALDH1B1 may induce the EMT phenotype through the ZEB1 cascade pathway (↑ ZEB1 → ↓ E-cadherin) in HT29 cells. ZEB1 is a transcription factor that is associated with development and cell differentiation, while it negatively regulates the transcriptional levels of *E-cadherin* through the binding to its promoter [65]. More specifically, ZEB1 interacts with the BRG1 protein and represses the promoter of *E-cadherin* [65]. Our findings are in agreement with previous experimental data indicating that ALDH_bright_ MKN-45 and SGC-7901 cells exhibited increased levels of *vimentin* and *SNAIL*, resulting in the downregulation of *E-cadherin* and, therefore, the acquisition of an EMT phenotype [2], during which, cells have increased survival, decreased proliferative rates and altered cytoskeletal morphology [44,66].

Finally, we studied the potential migratory effect of ALDH1B1 in HT29 cells by employing the scratch assay. Although migration and invasion are separated terms in cell biology, cell migration underlies tumor invasion. In our study, we focused on the collective migration mode, which contributes to tumor progression through local invasion [67,68]. HT29/ALDH1B1 cells appeared to migrate faster than mock control cells, demonstrating that ALDH1B1 expression increased the migration effect. Other ALDH members have also been implicated in the cell migration process. Moreb et al. demonstrated that the knockdown of ALDH1A1 and ALDH3A1 resulted in a decreased migration potential of A549 cells [69]. However, Croker et al. reported on the differential regulation of ALDH1A1 and ALDH1A3 on cell migration. Although ALDH1A1 knockdown led to the decreased migration of MDA-MB-468 and SUM159 cell migration, ALDH1A3 knockdown enhanced the migratory potential of these cells [70]. Although, at first glance, the slower proliferation rate of ALHD1B1-overexpressing cells appears to be contradictory in relation to their faster migration rate, they may be two sides of the same coin [71]. There are several findings supporting that an increase in cell proliferation is critical for the initiation and maintenance of a tumor, but on the other hand, growth inhibition could ultimately be important for the survival of carcinoma cells, leading to the development of a more malignant cancer phenotype. The YB-1 protein is frequently observed elevated in human cancers and has been associated with reduced proliferation in disseminated mesenchymal-like breast carcinoma cells. Similarly, YB-1 was reported to reduce cell proliferation and induce EMT in breast cancer cell lines, leading to increased mobility and invasiveness and an enhanced ability of the cells to survive in anchorage-independent conditions. A proliferation blockade induced by the overexpression of the cyclin-dependent kinase inhibitors p16(INK4a), p21(Cip1) or p27(Kip1) was also shown to be advantageous for the survival of normal and breast cancer cells under anchorage-independent conditions [72].

EMT induction is related to CSC properties such as chemoresistance [73], resulting in the decrease of cell proliferation [45] and metastasis [44]. In general, tumor cells are highly proliferative, but they proliferate at slower rates at the invasion stage and stop dividing at the migration stage during embryonic development and angiogenesis [44]. Recent studies showed that, during the metastatic process, which is strongly associated with CSCs [74], cells exhibited lower expression levels of proliferating cell nuclear antigen (PCNA) [75] and, therefore, possess a slower proliferation potential [76]. It is tempting to speculate that ALDH1B1 is correlated with the EMT process and/or with CSC plasticity, driving cells to migrate to other sites and, thus, regulating their transformation between the stem and non-stem cell stage [63].

In conclusion, HT29 ALDH1B1-overexpressing cells exhibited altered cell morphology; decreased proliferation and clonogenicity; p53-dependent G2/M cell cycle growth arrest and enhanced chemoresistance against doxorubicin, 5-FU and etoposide. On the other hand, ALDH1B1-overexpressing cells demonstrated increased migratory potential, most probably through the acquisition of an EMT phenotype via the upregulation of the ZEB1-dependent pathway. Overall, our findings enhance our understanding on the underlined role of ALDH1B1 in cancer progression.

## Figures and Tables

**Table 1:** Primers used for real-time PCR comparative quantification.

Gene	Forward Primer	Reverse Primer
*β-actin*	GCGCGGCTACAGCTTCA	CTTAATGTCACGCACGATTTCC
*ALDH1B1*	AGCCTCTGTTCAAGTTCAAG	CCTTAAAGCCTCCGAATGG
*E-cadherin*	TACACTGCCCAGGAGCCAGA	TGGCACCAGTGTCCGGATTA
*SNAI1*	ACTATGCCGCGCTCTTTCCT	GGTGGGGTTGAGGATCTCCG
*SNAI2*	CTACAGCGAACTGGACACAC	TGTGGTATGACAGGCATGGAG
*vimentin*	TGAGTACCGGAGACAGGTGCAG	TAGCAGCTTCAACGGCAAAGTTC
*ZEB1*	CGAGTCAGATGCAGAAAATGAGCAA	ACCCAGACTGCGTCACATGTCTT
*ZEB2*	ACTATGGGGCCAGAAGCCAC	CTGCATGACCATCGCGTTCC
*TWIST1*	AGCTACGCCTTCTCGGTCTG	TGGGAATCACTGTCCACGGG
*N-cadherin*	CCTACTGGACGGTTCGCCAT	GTTGCAGTTGACTGAGGCGG

**Table 2:** Median fluorescence intensity for forward scatter (FSC) and side scatter (SSC) parameters.

	Parameter	HT29/mock	HT29/ALDH1B1	Statistical Significance
Median Fluorescence Intensity	FSC	247,552 ± 13,408.88 ^†^	292,973.2 ± 37,072.24 ^†^	*
	SSC	208,992 ± 20,580.28 ^†^	167,270.4 ± 19,655.07 ^†^	*

^†^ Results are expressed as mean ± SD of three independent experiments. * *p* < 0.05.

**Table 3:** Doubling time (tD) and growth rates of the HT29 isogenic cell line pair.

Sample	Doubling Time (tD) (h)	Growth Rates (GR)
HT29/mock	24.16 ± 0.05	0.029 ± 0.0004
HT29/ALDH1B1	26.75 ± 0.02	0.026 ± 0.0007

**Table 4:** Median fluorescence intensity for p21.

	Time (h)	HT29/mock	HT29/ALDH1B1	Statistical Significance
Median Fluorescence Intensity	0	90 ± 3.68 ^†^	142 ± 5.66 ^†^	*
	24	92.93 ± 2.08 ^†^	168.67 ± 10.01 ^†^	**

^†^ Results are expressed as mean ± SD of three independent experiments. * *p* < 0.05 and ** *p* < 0.01.

**Table 5:** EC_50_ values of doxorubicin (72 h), fluorouracil (5-FU) (72 h) and etoposide (48 h).

Sample	Doxorubicin (μM) (72 h)	5-FU (μM) (72 h)	Etoposide (μM) (48 h)
HT29/mock	0.59 ± 0.079 ^†^	0.92 ± 0.15 ^†^	67.85 ± 1.67 ^†^
HT29/ALDH1B1	1.073 ± 0.13 ^†^	1.79 ± 0.056 ^†^	92.47 ± 0.65 ^†^
Statistical significance	**	***	**

^†^ Results are expressed as mean ± SD of three independent experiments. ** *p* < 0.01 and *** *p* < 0.001.

**Table 6:** Percentage (%) of unstained and propidium iodide (PI)-stained cells under normal and etoposide conditions.

Sample		Untreated	Etoposide 20 μM	Etoposide 25 μM
HT29/mock	PI−	77 ± 1.91 ^†^	71.2 ± 1.37 ^†^	63.3 ± 2.33 ^†^
	PI+	23 ± 2.16 ^†^	28.8 ± 0.95 ^†^	36.7 ± 1.44 ^†^
HT29/ALDH1B1	PI−	80.5 ± 1.66 ^†^	76.6 ± 1.14 ^†^	71.1 ± 1.77 ^†^
	PI+	19.5 ± 1.31 ^†^	23.4 ± 2.03 ^†^	28.9 ± 1.82 ^†^
Statistical significance	PI− vs. PI−	-	*	***
	PI+ vs. PI+	-	*	***

^†^ Results are expressed as mean ± SD of three independent experiments. * *p* < 0.05 and *** *p* < 0.001.

## Supplementary Materials

The following are available online at https://www.mdpi.com/2227-9059/9/1/44/s1, Figure S1: Effect of ALDH1B1 overexpression in Caco2 cells. Table S1: Median fluorescence intensity for FSC and SSC parameters.
