# Supplementary material for: Aldehyde Dehydrogenase 1B1 Is Associated with Altered Cell Morphology, Proliferation, Migration and Chemosensitivity in Human Colorectal Adenocarcinoma Cells
Source: Biomedicines. 2021 Jan 6;9(1):44. doi: 10.3390/biomedicines9010044 (PMC7825346; doi:10.3390/biomedicines9010044)
Supplement: Supplementary file 1 [file biomedicines-09-00044-s001.pdf]

Aldehyde Dehydrogenase 1B1 Is Associated with Altered Cell Morphology, Proliferation, Migration and Chemosensitivity in Human Colorectal Adenocarcinoma Cells

Ilias Tsochantaridis, Angelos Roupas, Georgia-Persephoni Voulgaridou, Alexandra Giatromanolaki, Michael I. Koukourakis, Mihalis I. Panayiotidis and Aglaia Pappa

Supplementary Material

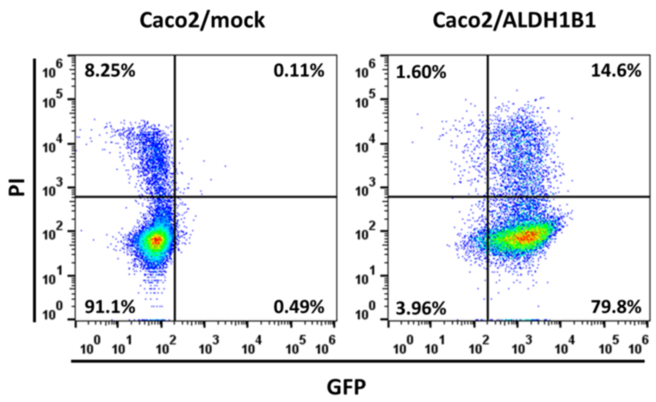

(a)

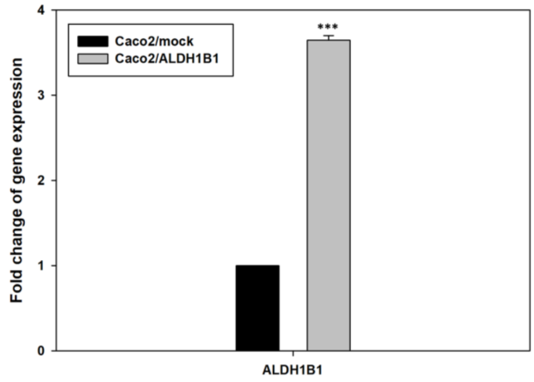

(b)

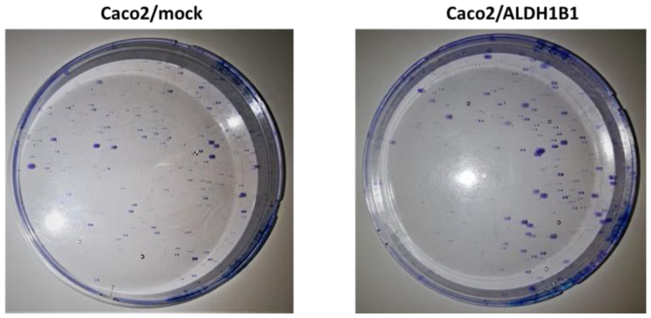

(c)

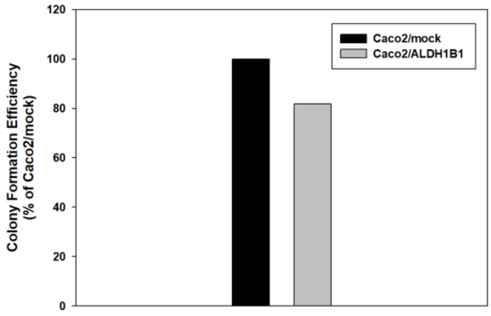

Sample	Colonies (> 50cells)
Caco2/mock	198
Caco2/ALDH1B1	162

(d)

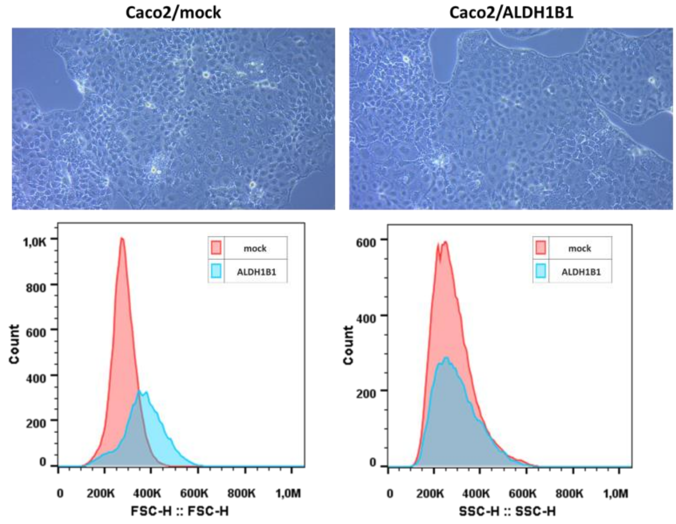

(**e**)

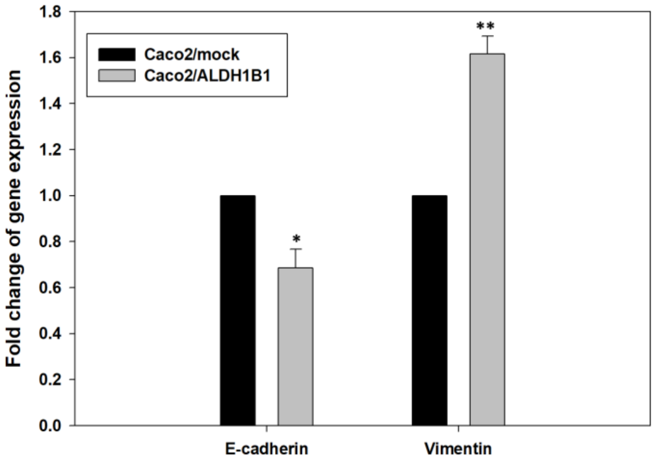

(**f**)

Figure S1. Effect of ALDH1B1 overexpression in CaCo2 cells. (**a**) Evaluation of GPF+ cells (green) in Caco2/mock and Caco2/ALDH1B1 by flow cytometry analysis. (**b**) *ALDH1B1* gene expression levels detected by real-time PCR in Caco2/mock and Caco2/ALDH1B1 cells. (**c**) Representative plates of colony formation efficiency of Caco2/mock and Caco2/ALDH1B1 cells. Cells (1000) were seeded in 10 cm culture plates and observed for colony formation approximately 20 days following their plating. Then, cells were stained with crystal violet. (**d**) Quantitative assessment of colony formation efficiency. (**e**) Expression of ALDH1B1 triggers significant morphological alterations in Caco2 cells. ALDH1B1-expressing cells have different size compared to mock control cells. At least 20,000 events were analyzed through flow cytometry and the median fluorescence intensity of FCS and SSC parameters was determined in both cell lines (Table S1). (**f**) Expression of ALDH1B1 induces EMT in Caco2 cells. ALDH1B1 induce the up-regulation of *vimentin* and the down-regulation of *E-cadherin* transcriptional expression levels in the Caco2 isogenic cell line pair. The comparative quantification ΔΔCt method was utilized for analyzing the fold change in gene expression levels. β-actin gene was used as endogenous control for the normalization of samples. Results are shown as mean ± S.D. At least three independent experiments were performed under each experimental condition. *p<0.05, **p<0.01.

Table S1. Median fluorescence intensity for FSC and SSC parameters.

	Parameter	Caco2/mock	Caco2/ALDH1B1	Statistical significance
Median Fluorescence Intensity	FSC	278,954.7 ± 5,516.39 †	344,405.3 ± 21,141.84 †	*
	SSC	272,810.7 ± 6,508.31 †	298,213.3 ± 16,769.65 †	-

† Results are expressed as mean ±S.D. of three independent experiments. * p<0.05.
